# Mutagenic and Cytotoxic Properties of Oxidation Products of 5-Methylcytosine Revealed by Next-Generation Sequencing

**DOI:** 10.1371/journal.pone.0072993

**Published:** 2013-09-16

**Authors:** Xi-Wen Xing, Yu-Li Liu, Mario Vargas, Yinsheng Wang, Yu-Qi Feng, Xiang Zhou, Bi-Feng Yuan

**Affiliations:** 1 Key Laboratory of Analytical Chemistry for Biology and Medicine (Ministry of Education), Department of Chemistry, Wuhan University, Wuhan, P.R. China; 2 Department of Chemistry, University of California Riverside, Riverside, California, United States of America; University of Massachusetts Medical School, United States of America

## Abstract

5-methylcytosine (5-mC) can be sequentially oxidized to 5-hydroxymethylcytosine (5-hmC), 5-formylcytosine (5-foC), and finally to 5-carboxylcytosine (5-caC), which is thought to function in active DNA cytosine demethylation in mammals. Although the roles of 5-mC in epigenetic regulation of gene expression are well established, the effects of 5-hmC, 5-foC and 5-caC on DNA replication remain unclear. Here we report a systematic study on how these cytosine derivatives (5-hmC, 5-foC and 5-caC) perturb the efficiency and accuracy of DNA replication using shuttle vector technology in conjugation with next-g sequencing. Our results demonstrated that, in *Escherichia coli* cells, all the cytosine derivatives could induce CT transition mutation at frequencies of 0.17%–1.12%, though no effect on replication efficiency was observed. These findings provide an important new insight on the potential mutagenic properties of cytosine derivatives occurring as the intermediates of DNA demethylation.

## Introduction

Every single cell in a living organism carries the genome, which functions for the storage, replication and transmission of the genetic information. In addition to this basic hereditary genetic information, DNA contains epigenetic modifications that are present in the genomes [1]. Cytosine methylation (5-methylcytosine, 5-mC) at CpG dinucleotide site is the best-characterized epigenetic mark involved in regulating many cellular processes, including embryogenesis, regulation of gene expression, genomic imprinting and X-chromosome inactivation [2]. Consistent with these important roles, a variety of human diseases have been found to be associated with aberrant DNA methylation [3], [4].

**Figure 1:**
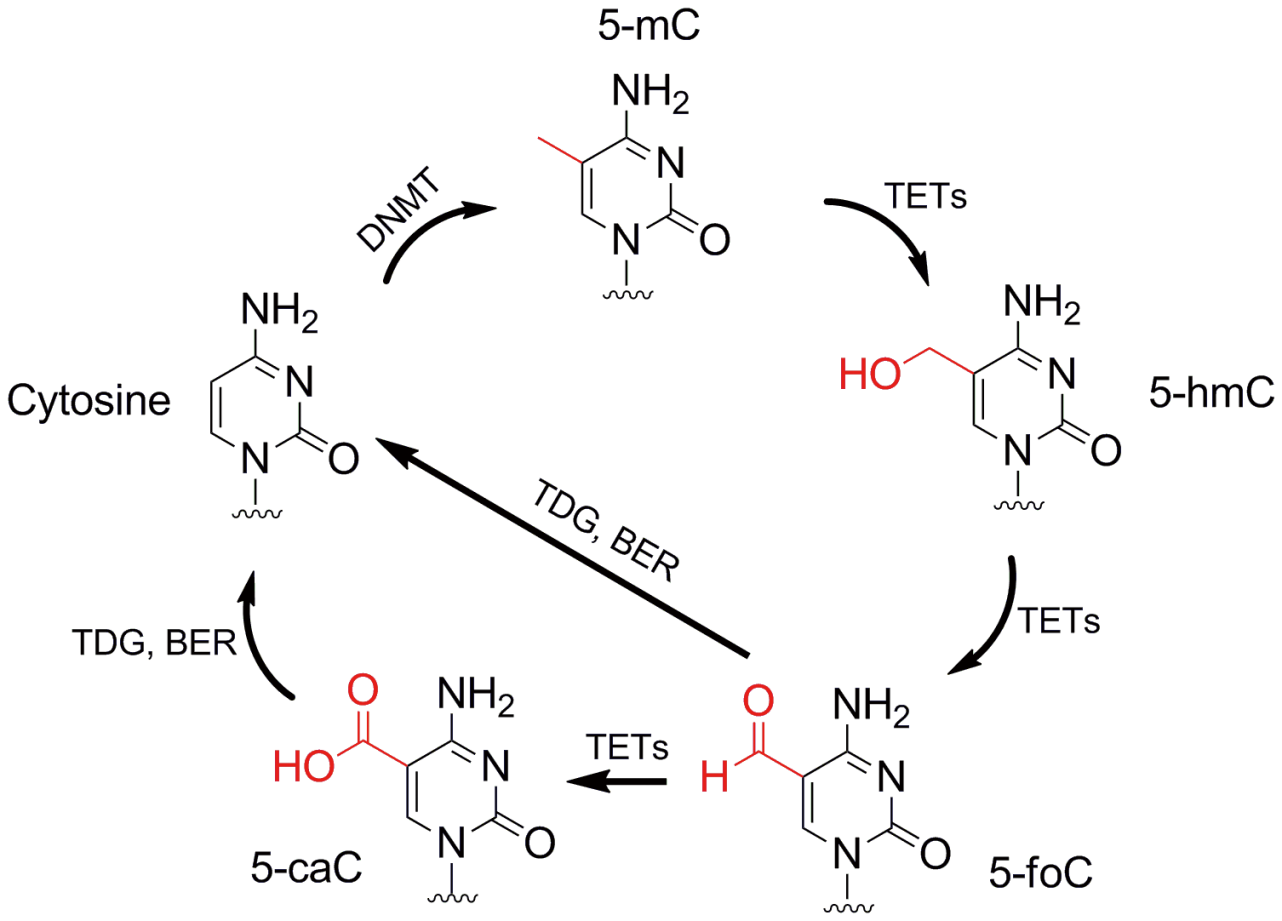
The structures of the cytosine derivatives. 5-mC modification is catalyzed by DNA methyltransferase (DNMT). 5-mC can be demethylated through the oxidation of 5-mC by TET proteins to produce 5-hmC, 5-fC and 5-caC. TDG, thymine-DNA glycosylase; BER, base-excision repair.

DNA methylation undergoes dynamic changes and is reversible in a genome-wide or locus-specific manner [5]; however, the mechanisms of active DNA demethylation in mammals have been a matter of debate for many years [6]. Recent studies showed that Ten–Eleven Translocation (TET) proteins are capable of catalyzing the sequential oxidation of 5-mC to 5-hydroxymethylcytosine (5-hmC), 5-formylcytosine (5-foC), and finally to 5-carboxylcytosine (5-caC) (Figure 1) [7], [8], [9], [10]. Follow-up report revealed that 5-caC can be further recognized and cleaved by thymine-DNA glycosylase (TDG) and then the unmethylated cytosine can be restored via base-excision repair pathway [11]. Therefore, active DNA demethylation may be achieved through a multi-step oxidation of 5-mC with the generation of three intermediates, 5-hmC, 5-foC and 5-caC (Figure 1).

5-hmC plays important roles on cellular differentiation [10] and epigenetic regulation [12], [13] and itself may serve as an epigenetic mark with regulatory functions aside from being an intermediate of active DNA cytosine demethylation [14]. In addition, previous reports together with our recent study revealed that 5-hmC content in tumor tissues were significantly lower than that in healthy control and tumor adjacent tissues [15], [16], [17], [18], suggesting that 5-hmC in genomic DNA might also be associated with tumor development. 5-hmC content in mammalian tissues and cells varies from 0.009% to 1.03% of cytosine (molar ratio of 5-hmC/cytosine or guanine) [19], [20], [21]. Although much less abundant than 5-hmC, 5-foC and 5-caC are present at a level of between 10^3^ and 10^5^ per human cell [20], [21], [22], [23], [24], which is comparable to (for 5-foC and 5-caC) or much higher (for 5-hmC) than the frequency of DNA damage induced by environmental and endogenous agents [25]. DNA damage frequently induces mutations in genome and compromises DNA replication and transcription. Therefore, the presence of the oxidation products of 5-methylcytosine (5-hmC, 5-foC and 5-caC) in the genome raises an intriguing question on how these cytosine derivatives may affect DNA replication. To this end, here we systematically investigated the *in-vivo* replication of 5-hmC, 5-foC and 5-caC by using our recently developed method, i.e. shuttle vector technology in conjunction with the next-generation sequencing (NGS) [26]. In the current study, we examined how these cytosine derivatives perturb the efficiency and accuracy of DNA replication in *Escherichia coli* (*E. coli*) cells. Our results demonstrated that all the cytosine derivatives of 5-hmC, 5-foC and 5-caC could induce CT transition mutation, but none of them inhibit DNA replication in *E. coli* cells.

## Materials and Methods

### Chemicals and Cell Strains

Modified and unmodified oligodeoxyribonucleotides (ODNs) used in this study were all purchased from TaKaRa Biotechnology (Dalian, China). The sequences of 27mer 5-hmC-, 5-foC- and 5-caC-containing ODNs were listed in Table 1. The identities of the modified ODNs were confirmed by Matrix-Assisted Laser Desorption/ Ionization – Time of Flight Mass Spectrometry (MALDI-TOF MS) (Figure S1). To differentiate the progeny vectors for individual cytosine derivative after *in-vivo* replication, a trinucleotide barcode was incorporated into the 27mer ODNs (Table 1, and barcode sequences were underlined). To examine the influence of sequence contexts on the replication of cytosine derivatives, we employed the ODNs with a 2′-deoxyguanosine (XG sequences) or 2′-deoxyadenosine (XA sequences) as the neighboring 3′ nucleoside (Table 1).

**Table 1:** The sequences of the 27mer cytosine derivative-containing and the control ODNs used for replication studies.

Name of ODNs	Sequences
5-hmC-XG	5′-GAGTCGCGACCCATGGG**XG**CCGAATTC**-3′**
5-foC-XG	5′-GAGTCGCGTGCCATGGG**XG**CCGAATTC-3′
5-caC-XG	5′-GAGTCGCATACCATGGG**XG**CCGAATTC-3′
control-CG	5′-GAGTCGCGCTCCATGGG**CG**CCGAATTC-3′
5-hmC-XA	5′-GAGTCGCTGACCATGGG**XA**CCGAATTC-3′
5-foC-XA	**5′-**GAGTCGCTCCCCATGGG**XA**CCGAATTC**-3′**
5-caC-XA	5′-GAGTCGCTAGCCATGGG**XA**CCGAATTC-3′
control-CA	5′-GAGTCGCCTCCCATGGG**CA**CCGAATTC-3′

‘X’ designates modified cytosine and the barcode is underlined in each 27mer sequence.

All enzymes were obtained from TaKaRa Biotechnology. Chemicals unless otherwise noted were obtained from Sigma-Aldrich (St. Louis, MO). M13mp7 (L2) and wild-type AB1157 *E. coli* strains were kindly provided by Prof. John M. Essigmann, and polymerase-deficient AB1157 strains [*Δpol B1*::spec (pol II-deficient), *ΔdinB* (pol IV-deficient), *ΔumuC*::kan (pol V-deficient) and *ΔumuC*::kan *ΔdinB* (pol IV, pol V-double knockout)] were generously provided by Prof. Graham C. Walker [27].

### Construction of ssM13 Genomes Harboring a Site-specifically Inserted Cytosine Derivative

The M13mp7 (L2) viral genomes, either control or carrying a site-specifically inserted cytosine derivative, were prepared following the previously described procedures [28]. Briefly, 20 pmol of ssM13mp7 (L2) was digested with 40 U EcoRI at 23°C for 8 h to linearize the vector. Two scaffolds, 5′-GCGACTCCACTGAATCATGGTCATAGCTTTC-3′ and 5′-GTAAAACGACGGCCAGTGAATTGAATTCGG-3′ (25 pmol), each spanning one end of the cleaved vector and the modified ODN insert, were annealed with the linearized vector. The 27mer modified ODN insert (30 pmol, Table 1) was 5′-phosphorylated with T4 polynucleotide kinase followed by ligating to the above vector by using T4 DNA ligase in the presence of the two scaffolds at 16°C for 8 h. T4 DNA polymerase (20 U) was subsequently added and the resulting mixture was incubated at 37°C for 4 h to degrade the scaffolds and residual unligated vector. The reaction mixture was purified with DNA Clean-up kit (Cycle-Pure Kit, Omega, Guangzhou, China) to obtain the cytosine derivative-containing vector.

### Transfection of *E. coli* Cells with ssM13 Vectors Containing a 5-hmC, 5-foC or 5-caC

Desalted 5-hmC, 5-foC and 5-caC-containing as well as control M13 genomes were mixed at 1∶1 ratio (25 fmol each) and transfected into wild-type AB1157 *E. coli* cells and the isogenic *E. coli* cells that are deficient in pol II, pol IV, pol V, or both pol IV and pol V. The electrocompetent cells were prepared following the previously published procedures [29]. After transfection, the *E. coli* cells were grown in LB culture at 37°C for 6 h, after which the phage was recovered from the supernatant by centrifugation at 13,000 rpm for 5 min. The resulting phage was further amplified in SCS110 *E. coli* cells to increase the progeny/cytosine derivative-genome ratio [28]. The phage recovered from the supernatant was passed through a QIAprep Spin M13 column (Qiagen) to isolate the ssM13 DNA.

### Generation of Sequencing Library and Determination of the Bypass Efficiency and Mutation Frequency by NGS

The sequencing library was generated using NEBNext® DNA Sample Prep Master Mix Set 1 (New England Biolabs, Ipswich, MA, Figure S2). Briefly, 15 sets of primers each housing a unique trinucleotide barcode (Table S1), which designated host cell lines or individual biological replicates, were employed to generate PCR products from the progeny vectors. PCR amplification of the region of interest in the resulting progeny genome was performed using Phusion high-fidelity DNA polymerase (New England Biolabs) and running at 98°C for 60 s and 15 cycles at 98°C for 10 s, 44°C for 30 s and 72°C for 5 s, with a final extension at 72°C for 5 min. The 15 sets of PCR products were purified by QIAquick Nucleotide Removal Kit (Qiagen) and then mixed at equal amounts. The PCR mixture was phosphorylated at 5′ end using T4 polynucleotide kinase. A single ‘A’ nucleotide was added to the 3′ end of the PCR products and the resulting purified PCR mixture was ligated to two PE Adapters (Table S1). The ligation products were further amplified using PE PCR primers (Table S1) under the same conditions as described above. The resulting PCR products (172 bp) were gel-purified and subjected to NGS using Illumina Genome Analyzer IIe system (Illumina, San Diego, CA).

After obtaining the raw sequencing data, the reads of low quality or with undefined nucleobase in sequence were filtered and removed from the raw reads. The distributions of barcodes in the resulting filtered reads and the nucleobase (A, T, C or G) frequencies at the specific cytosine derivative site were analyzed according to our previously reported method [26]. The bypass efficiency was calculated using the following formula, %bypass =  total number of reads from cytosine derivative genome/total number of reads from control genome. The mutation frequencies were calculated using the following formula, %mutation =  total number of reads of A, T, C or G at original cytosine derivative site from cytosine derivative genome/total number of reads from cytosine derivative genome.

## Results

**Figure 2:**
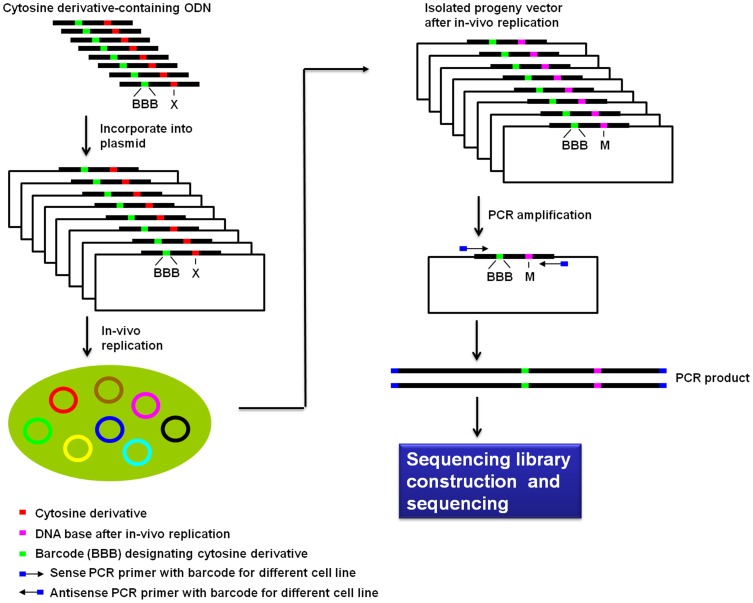
A schematic diagram outlining the experimental procedures. The 27mer cytosine derivative-containing ODNs with barcodes were ligated to the EcoR I-linearized M13 vector, mixed at equal amounts and subjected to *in-vivo* replication. The harvested M13 progenies were amplified with barcoded PCR primers, and equal amounts of PCR products from different cell lines were mixed and subjected to NGS library construction and sequencing.

Our strategy for high-throughput mutagenesis study involves the use of a combination of NGS with shuttle vector technology, as depicted in Figure 2. Following previously published procedures [30], [31], [32], we constructed the single-stranded (ss) M13 shuttle vectors carrying structurally defined cytosine derivative at a specific site. Six cytosine derivative-bearing and two control M13 genomes were mixed together and transfected into *E. coli* cells to examine the *in-vivo* mutagenic and cytotoxic properties. To illustrate the roles of various translesion synthesis DNA polymerases in bypassing these cytosine derivatives *in vivo*, we employed wild-type AB1157 *E. coli* cells as well as the isogenic strains deficient in pol II, pol IV, pol V, or both pol IV and pol V as the host cells for the replication study. After *in-vivo* replication, the ssM13 progeny vectors were isolated. Fifteen pairs of barcoded primers (Table S1), which designated 15 distinct sets of progeny genomes arising from triplicate replication experiments in 5 different host cell lines, were employed to generate PCR products from the progeny vectors. The 15 sets of PCR products were then mixed at equal amounts and the resulting PCR product mixture was phosphorylated at the 5′ end, adenylated at the 3′ end, and ligated to PE Adapters 1 and 2 (Table S1). The ligation products were further amplified using PE PCR primers (Table S1), and the resulting PCR products were gel-purified and subjected to NGS analysis using Illumina Genome Analyzer IIe system. From the sequencing results, we determined the mutagenic and cytotoxic properties of cytosine derivatives in different bacterial hosts by interrogating the distribution of barcodes and nucleobase (A, T, C or G) frequencies at the specific site. In addition, the sequencing reads obtained for the cytosine derivative-containing genomes relative to control genomes allowed for the calculation of bypass efficiencies for the cytosine derivative.

**Figure 3:**
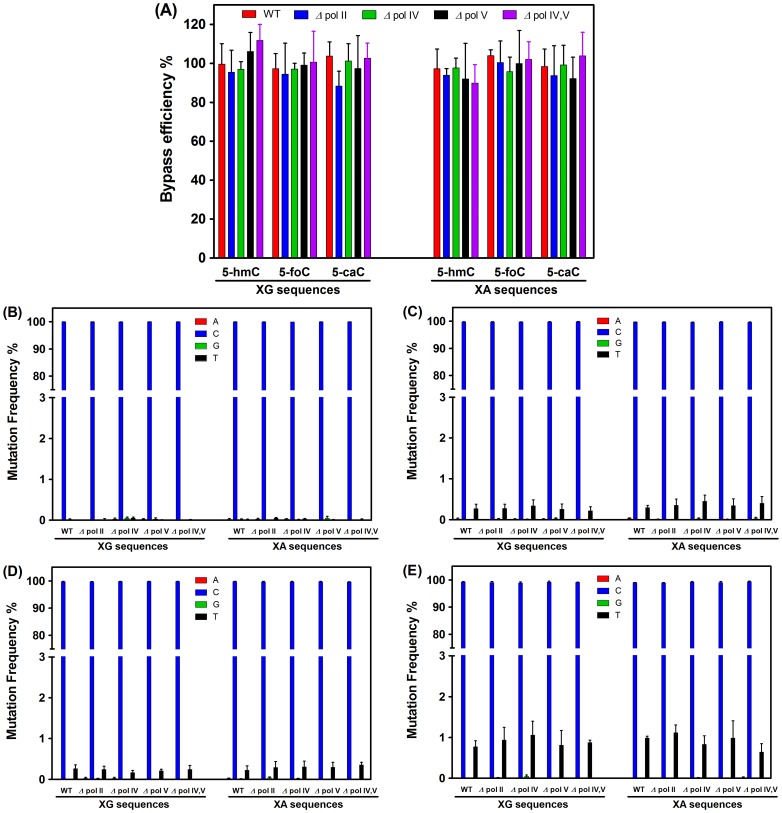
Bypass efficiencies and mutation frequencies of cytosine derivatives. (A) Bypass efficiencies of 5-hmC, 5-foC and 5-caC. (B–E) Mutation frequencies of control (B), 5-hmC (C), 5-foC (D) and 5-caC (E). The data represent the means and standard deviations of results from three independent experiments.

We obtained a total of 0.52 million valid sequencing reads for the replication products of these genomes. Table S2 and S3 show the number of reads obtained for replication products, which is much more than what can be achieved with traditional colony picking and Sanger sequencing method. The bypass efficiencies were calculated from the ratio of the total number of reads from cytosine derivative genome over the total number of reads from the control genome. It turned out that the bypass efficiencies of 5-hmC, 5-foC and 5-caC varied from ∼90% to 110% in wild-type AB1157 *E. coli* cells as well as in the isogenic strains deficient in pol II, pol IV, pol V, or both pol IV and pol V (Figure 3A), which suggested than these cytosine derivatives basically did not block DNA replication. The results from NGS data also allowed us to assess the mutation frequencies of cytosine derivatives in wild-type and bypass polymerase-deficient *E. coli* strains. The quantification data showed that all the cytosine derivatives of 5-hmC, 5-foC and 5-caC are mutagenic, with CT transition occurring at frequencies of 0.17%–1.12% and with 5-caC being the most mutagenic (0.65% to 1.12%) (Figure 3B–3E).

Our *in-vivo* replication study also revealed no significant difference of CT mutation between wild-type AB1157 and bypass polymerase-deficient *E. coli* strains for each cytosine derivative, suggesting that DNA pol II, pol IV or pol V may not be involved in the replicative bypass of these cytosine derivatives. It is possible that the bypass efficiencies and mutation frequencies of the cytosine derivatives may differ in different sequence contexts. Here we also assessed the effects of sequence context on DNA replication. The results demonstrate that the overall CT mutation induced by XG sequences is comparable to the mutation induced by XA sequences for each cytosine derivative (Figure 3), suggesting a lack of sequence context effect.

## Discussion

Recently, it was discovered that the epigenetic mark of 5-mC can be further sequentially oxidized to 5-hmC, 5-foC and 5-caC, which are present in substantial levels in the genome of cells [20], [21], [22], [23], [24]. These oxidation products of 5-mC potentially could stimulate cellular mutagenic events due to their uncanonical nucleobases. In this study we systematically explored the *in-vivo* mutagenicity and cytotoxicity of 5-hmC, 5-foC and 5-caC.

Previous *in-vitro* experiments (primer extension assay) showed that 5-foC was able to induce slight CT transition mutation at frequency of 1%–2% using either high fidelity polymerase Klenow fragment (exo^−^) or low fidelity polymerase η and κ [33]. The frequency of CT mutation induced by 5-foC of the *in-vitro* experiments is comparable with our *in-vivo* assay. A recent study also revealed that 5-foC and 5-caC affect the substrate specificities and transcriptional fidelity of RNA polymerase II transcription [24]. The substitution of cytosine with 5-foC in DNA reduces the fidelity of nucleotide incorporation by a factor of ∼30 during transcription [24]. Human genomic mutation occurs at a frequency of ∼ 1.1–2.5×10^−8^ per base [34], [35]. Considering the contents of 5-hmC, 5-foC and 5-caC in cellular DNA, the mutations induced by these cytosine derivatives can be a relative large number compared to the natural mutation frequency of nucleobases. The mutagenic properties of cytosine derivatives induced in both replication and transcription steps may therefore compromise the genome fidelity and finally jeopardize the physiological functions of cells. It is of note that we employed *E. coli* cells as the host cells for the current study, which may not faithfully reflect the situation in mammalian cells. Further exploration of the replication of cytosine derivatives in mammalian cells is necessary for understanding their mutagenic and cytotoxic properties in mammalian cells. Nevertheless, the replicating properties of cytosine derivatives demonstrated in the current study, together with the previous report showing that 5-foC was mutagenic [33] as well as 5-foC and 5-caC reduced transcriptional fidelity [24], provide new insights on the mutagenic properties of the intermediates produced during active DNA cytosine demethylation.

A relative high error rate (1.2% for the control genome) was observed in our previous study, which is partially attributed to the sequencing error produced at the barcode sites [26]. Therefore, modified nucleobases with an induced mutation frequency that is <3–4% could not be accurately assessed. To circumvent this problem, we employed trinucleotide barcodes for the present study. The error rate was found to be lower than 0.05% with the use of trinucleotide barcodes, which is much lower than that obtained with dinucleotide barcodes; therefore, the method is capable of evaluating extremely low frequencies of mutations induced by the modified nucleobases.

Taken together, our current study demonstrated that the oxidized 5-mC derivatives can induce mutation, but they did not affect the replication efficiency in *E. coli* cells. These findings provide an important new perspective on the potential mutagenic properties of the cytosine derivatives occurring as the intermediates of DNA demethylation.

## Supporting Information

Figure S1 **Negative-ion MALDI-TOF mass spectra of the 27mer cytosine derivative-containing ODNs.**
(DOC)

Figure S2 **NGS sample preparation workflow.**
(DOC)

Table S1 **PCR primers with trinucleotide barcodes at 5′ end, PE adapters, PE PCR primers and NGS sequencing primer.**
(DOC)

Table S2 **The number of reads obtained by NGS for XG sequences.**
(DOC)

Table S3 **The number of reads obtained by NGS for XA sequences.**
(DOC)

## References

[1] Ndlovu MN, Denis H, Fuks F (2011) Exposing the DNA methylome iceberg. Trends Biochem Sci 36: 381–387. 21497094 10.1016/j.tibs.2011.03.002

[2] Bird A (2002) DNA methylation patterns and epigenetic memory. Genes Dev 16: 6–21. 11782440 10.1101/gad.947102

[3] Rottach A, Leonhardt H, Spada F (2009) DNA methylation-mediated epigenetic control. J Cell Biochem 108: 43–51. 19565567 10.1002/jcb.22253

[4] Robertson KD (2005) DNA methylation and human disease. Nat Rev Genet 6: 597–610. 16136652 10.1038/nrg1655

[5] Hackett JA, Zylicz JJ, Surani MA (2012) Parallel mechanisms of epigenetic reprogramming in the germline. Trends Genet 28: 164–174. 22386917 10.1016/j.tig.2012.01.005

[6] Wu SC, Zhang Y (2010) Active DNA demethylation: many roads lead to Rome. Nat Rev Mol Cell Biol 11: 607–620. 20683471 10.1038/nrm2950 PMC3711520

[7] Tahiliani M, Koh KP, Shen Y, Pastor WA, Bandukwala H, et al (2009) Conversion of 5-methylcytosine to 5-hydroxymethylcytosine in mammalian DNA by MLL partner TET1. Science 324: 930–935. 19372391 10.1126/science.1170116 PMC2715015

[8] Kriaucionis S, Heintz N (2009) The nuclear DNA base 5-hydroxymethylcytosine is present in Purkinje neurons and the brain. Science 324: 929–930. 19372393 10.1126/science.1169786 PMC3263819

[9] Ito S, Shen L, Dai Q, Wu SC, Collins LB, et al (2011) Tet Proteins Can Convert 5-Methylcytosine to 5-Formylcytosine and 5-Carboxylcytosine. Science 333: 1300–1303. 21778364 10.1126/science.1210597 PMC3495246

[10] Ito S, D'Alessio AC, Taranova OV, Hong K, Sowers LC, et al (2010) Role of Tet proteins in 5mC to 5hmC conversion, ES-cell self-renewal and inner cell mass specification. Nature 466: 1129–1133. 20639862 10.1038/nature09303 PMC3491567

[11] He YF, Li BZ, Li Z, Liu P, Wang Y, et al (2011) Tet-mediated formation of 5-carboxylcytosine and its excision by TDG in mammalian DNA. Science 333: 1303–1307. 21817016 10.1126/science.1210944 PMC3462231

[12] Kriukiene E, Liutkeviciute Z, Klimasauskas S (2012) 5-Hydroxymethylcytosine – the elusive epigenetic mark in mammalian DNA. Chem Soc Rev 41: 6916–6930. 22842880 10.1039/c2cs35104h PMC3467341

[13] Branco MR, Ficz G, Reik W (2012) Uncovering the role of 5-hydroxymethylcytosine in the epigenome. Nat Rev Genet 13: 7–13. 10.1038/nrg3080 22083101

[14] Shen L, Zhang Y (2013) 5-Hydroxymethylcytosine: generation, fate, and genomic distribution. Curr Opin Cell Biol 25: 289–296. 23498661 10.1016/j.ceb.2013.02.017 PMC4060438

[15] Kudo Y, Tateishi K, Yamamoto K, Yamamoto S, Asaoka Y, et al (2012) Loss of 5-hydroxymethylcytosine is accompanied with malignant cellular transformation. Cancer Sci 103: 670–676. 22320381 10.1111/j.1349-7006.2012.02213.x PMC7659252

[16] Chen ML, Shen F, Huang W, Qi JH, Wang Y, et al (2013) Quantification of 5-Methylcytosine and 5-Hydroxymethylcytosine in Genomic DNA from Hepatocellular Carcinoma Tissues by Capillary Hydrophilic-Interaction Liquid Chromatography/ Quadrupole Time-of-Flight Mass Spectrometry. Clin Chem 59: 824–832. 23344498 10.1373/clinchem.2012.193938 PMC3773166

[17] Ko M, Huang Y, Jankowska AM, Pape UJ, Tahiliani M, et al (2010) Impaired hydroxylation of 5-methylcytosine in myeloid cancers with mutant TET2. Nature 468: 839–843. 21057493 10.1038/nature09586 PMC3003755

[18] Yang H, Liu Y, Bai F, Zhang JY, Ma SH, et al (2013) Tumor development is associated with decrease of TET gene expression and 5-methylcytosine hydroxylation. Oncogene 32: 663–669. 22391558 10.1038/onc.2012.67 PMC3897214

[19] Jin SG, Jiang Y, Qiu R, Rauch TA, Wang Y, et al (2011) 5-Hydroxymethylcytosine is strongly depleted in human cancers but its levels do not correlate with IDH1 mutations. Cancer Res 71: 7360–7365. 22052461 10.1158/0008-5472.CAN-11-2023 PMC3242933

[20] Ito S, Shen L, Dai Q, Wu SC, Collins LB, et al (2011) Tet proteins can convert 5-methylcytosine to 5-formylcytosine and 5-carboxylcytosine. Science 333: 1300–1303. 21778364 10.1126/science.1210597 PMC3495246

[21] Liu S, Wang J, Su Y, Guerrero C, Mitra D, et al (2013) Quantitative assessment of Tet-induced oxidation products of 5-methylcytosine in cellular and tissue DNA. Nucleic Acids Res 41: 6421–6429. 23658232 10.1093/nar/gkt360 PMC3711458

[22] Pfaffeneder T, Hackner B, Truss M, Munzel M, Muller M, et al (2011) The discovery of 5-formylcytosine in embryonic stem cell DNA. Angew Chem Int Ed 50: 7008–7012. 10.1002/anie.201103899 21721093

[23] Raiber EA, Beraldi D, Ficz G, Burgess HE, Branco MR, et al (2012) Genome-wide distribution of 5-formylcytosine in embryonic stem cells is associated with transcription and depends on thymine DNA glycosylase. Genome Biol 13: R69. 22902005 10.1186/gb-2012-13-8-r69 PMC3491369

[24] Kellinger MW, Song CX, Chong J, Lu XY, He C, et al (2012) 5-formylcytosine and 5-carboxylcytosine reduce the rate and substrate specificity of RNA polymerase II transcription. Nat Struct Mol Biol 19: 831–833. 22820989 10.1038/nsmb.2346 PMC3414690

[25] Lord CJ, Ashworth A (2012) The DNA damage response and cancer therapy. Nature 481: 287–294. 22258607 10.1038/nature10760

[26] Yuan B, Wang J, Cao H, Sun R, Wang Y (2011) High-throughput analysis of the mutagenic and cytotoxic properties of DNA lesions by next-generation sequencing. Nucleic Acids Res 39: 5945–5954. 21470959 10.1093/nar/gkr159 PMC3152323

[27] Jarosz DF, Beuning PJ, Cohen SE, Walker GC (2007) Y-family DNA polymerases in *Escherichia coli* . Trends Microbiol 15: 70–77. 17207624 10.1016/j.tim.2006.12.004

[28] Delaney JC, Essigmann JM (2006) Assays for determining lesion bypass efficiency and mutagenicity of site-specific DNA lesions *in vivo* . Methods Enzymol 408: 1–15. 16793359 10.1016/S0076-6879(06)08001-3

[29] Neeley WL, Delaney S, Alekseyev YO, Jarosz DF, Delaney JC, et al (2007) DNA polymerase V allows bypass of toxic guanine oxidation products *in vivo* . J Biol Chem 282: 12741–12748. 17322566 10.1074/jbc.M700575200

[30] Yuan B, Jiang Y, Wang Y (2010) Efficient formation of the tandem thymine glycol/8-oxo-7,8-dihydroguanine lesion in isolated DNA and the mutagenic and cytotoxic properties of the tandem lesions in *Escherichia coli* cells. Chem Res Toxicol 23: 11–19. 20014805 10.1021/tx9004264 PMC2807900

[31] Yuan B, Wang Y (2008) Mutagenic and cytotoxic properties of 6-thioguanine, *S* ^6^-methylthioguanine, and guanine-*S* ^6^-sulfonic acid. J Biol Chem 283: 23665–23670. 18591241 10.1074/jbc.M804047200 PMC3259751

[32] Yuan B, Cao H, Jiang Y, Hong H, Wang Y (2008) Efficient and accurate bypass of *N* ^2^-(1-carboxyethyl)-2'-deoxyguanosine by DinB DNA polymerase *in vitro* and *in vivo* . Proc Natl Acad Sci U S A 105: 8679–8684. 18562283 10.1073/pnas.0711546105 PMC2438377

[33] Munzel M, Lischke U, Stathis D, Pfaffeneder T, Gnerlich FA, et al (2011) Improved synthesis and mutagenicity of oligonucleotides containing 5-hydroxymethylcytosine, 5-formylcytosine and 5-carboxylcytosine. Chemistry 17: 13782–13788. 22069110 10.1002/chem.201102782

[34] Nachman MW, Crowell SL (2000) Estimate of the mutation rate per nucleotide in humans. Genetics 156: 297–304. 10978293 10.1093/genetics/156.1.297 PMC1461236

[35] Roach JC, Glusman G, Smit AF, Huff CD, Hubley R, et al (2010) Analysis of genetic inheritance in a family quartet by whole-genome sequencing. Science 328: 636–639. 20220176 10.1126/science.1186802 PMC3037280

