# Supplementary material for: Mutagenic and Cytotoxic Properties of Oxidation Products of 5-Methylcytosine Revealed by Next-Generation Sequencing
Source: PLoS One. 2013 Sep 16;8(9):e72993. doi: 10.1371/journal.pone.0072993 (PMC3774748; doi:10.1371/journal.pone.0072993)
Supplement: Figure S1 — Negative-ion MALDI-TOF mass spectra of the 27mer cytosine derivative-containing ODNs. (DOC) [file pone.0072993.s001.doc]

**Figure S1.** Negative-ion MALDI-TOF mass spectra of the 27mer cytosine derivative-containing ODNs.

|  |  |
| --- | --- |
|  |  |
|  |  |
