# Supplementary figures and images for: Mutagenic and Cytotoxic Properties of Oxidation Products of 5-Methylcytosine Revealed by Next-Generation Sequencing
Source: PLoS One. 2013 Sep 16;8(9):e72993. doi: 10.1371/journal.pone.0072993 (PMC3774748; doi:10.1371/journal.pone.0072993)

**Figure S2.** NGS sample preparation workflow.


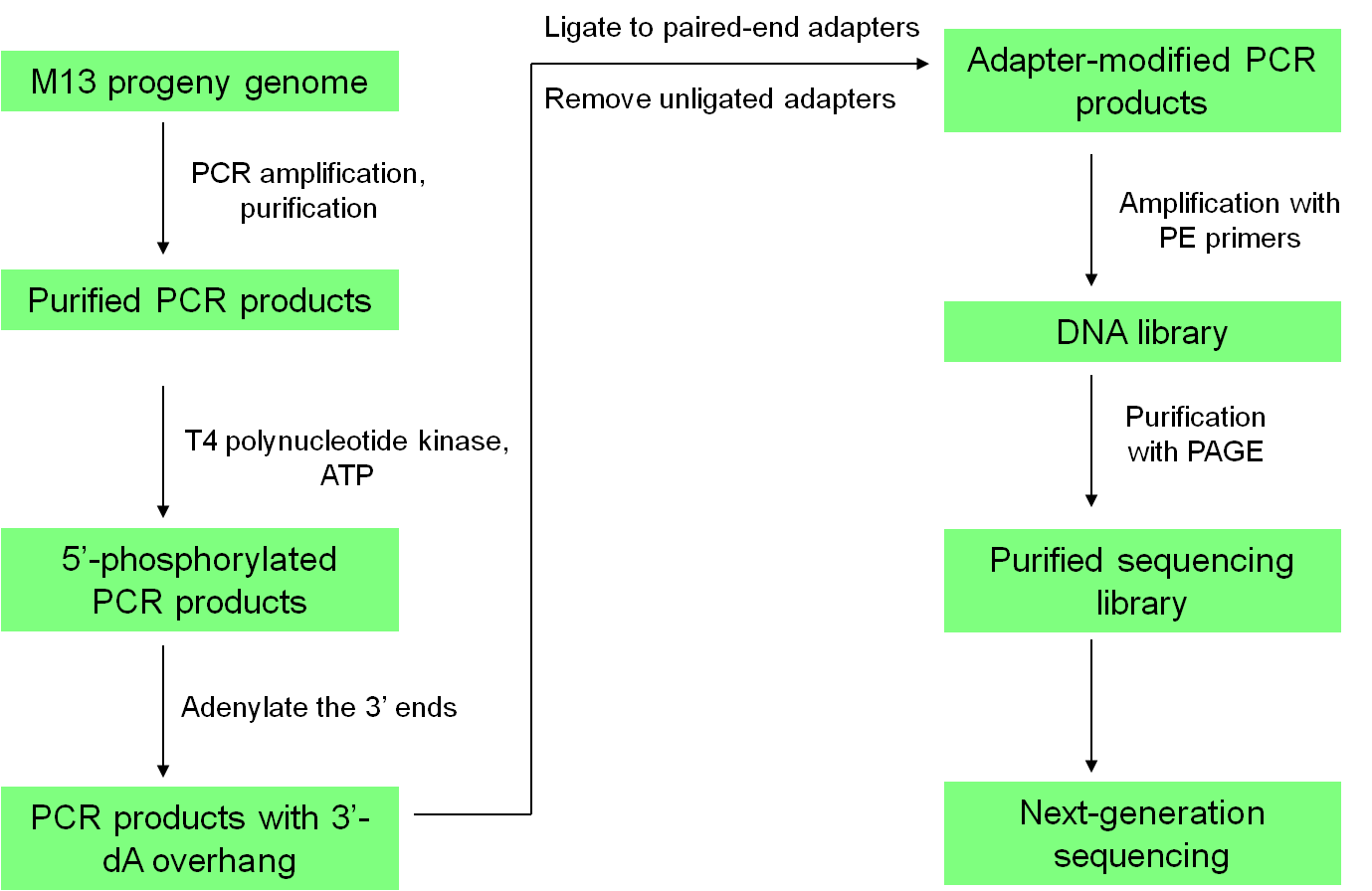

Supplement: Figure S2 — NGS sample preparation workflow. (DOC) [file pone.0072993.s002.doc]
