# Supplementary material for: Mutagenic and Cytotoxic Properties of Oxidation Products of 5-Methylcytosine Revealed by Next-Generation Sequencing
Source: PLoS One. 2013 Sep 16;8(9):e72993. doi: 10.1371/journal.pone.0072993 (PMC3774748; doi:10.1371/journal.pone.0072993)
Supplement: Table S1 — PCR primers with trinucleotide barcodes at 5′ end, PE adapters, PE PCR primers and NGS sequencing primer. (DOC) [file pone.0072993.s003.doc]

**Table S1**. PCR primers with trinucleotide barcodes at 5’ end, PE adapters, PE PCR primers and NGS sequencing primer. Trinucleotide barcodes highlighted in bold.

| 1. P1-S | 5’-**CAG**GATTCAGTGGAGTCGC-3’ |
| --- | --- |
| 2. P1-AS | 5’-**CAG**GCCAGTGAATTGAATTC-3’ |
| 3. P2-S | 5’-**CTG**GATTCAGTGGAGTCGC-3’ |
| 4. P2-AS | 5’-**CTG**GCCAGTGAATTGAATTC-3’ |
| 5. P3-S | 5’-**CAT**GATTCAGTGGAGTCGC-3’ |
| 6. P3-AS | 5’-**CAT**GCCAGTGAATTGAATTC-3’ |
| 7. P4-S | 5’-**TGA**GATTCAGTGGAGTCGC-3’ |
| 8. P4-AS | 5’-**TGA**GCCAGTGAATTGAATTC-3’ |
| 9. P5-S | 5’-**GCT**GATTCAGTGGAGTCGC-3’ |
| 10. P5-AS | 5’-**GCT**GCCAGTGAATTGAATTC-3’ |
| 11. P6-S | 5’-**TGC**GATTCAGTGGAGTCGC-3’ |
| 12. P6-AS | 5’-**TGC**GCCAGTGAATTGAATTC-3’ |
| 13. P7-S | 5’-**ACG**GATTCAGTGGAGTCGC-3’ |
| 14. P7-AS | 5’-**ACG**GCCAGTGAATTGAATTC-3’ |
| 15. P8-S | 5’-**AGT**GATTCAGTGGAGTCGC-3’ |
| 16. P8-AS | 5’-**AGT**GCCAGTGAATTGAATTC-3’ |
| 17. P9-S | 5’-**AGC**GATTCAGTGGAGTCGC-3’ |
| 18. P9-AS | 5’-**AGC**GCCAGTGAATTGAATTC-3’ |
| 19. P10-S | 5’-**GAC**GATTCAGTGGAGTCGC-3’ |
| 20. P10-AS | 5’-**GAC**GCCAGTGAATTGAATTC-3’ |
| 21. P11-S | 5’-**GTA**GATTCAGTGGAGTCGC-3’ |
| 22. P11-AS | 5’-**GTA**GCCAGTGAATTGAATTC-3’ |
| 23. P12-S | 5’-**GTC**GATTCAGTGGAGTCGC-3’ |
| 24. P12-AS | 5’-**GTC**GCCAGTGAATTGAATTC-3’ |
| 25. P13-S | 5’-**CGC**GATTCAGTGGAGTCGC-3’ |
| 26. P13-AS | 5’-**CGC**GCCAGTGAATTGAATTC-3’ |
| 27. P14-S | 5’-**GCA**GATTCAGTGGAGTCGC-3’ |
| 28. P14-AS | 5’-**GCA**GCCAGTGAATTGAATTC-3’ |
| 29. P15-S | 5’-**TAC**GATTCAGTGGAGTCGC-3’ |
| 30. P15-AS | 5’-**TAC**GCCAGTGAATTGAATTC-3’ |
| 31. PE Adapter1 | 5’-ACACTCTTTCCCTACACGACGCTCTTCCGATCT-3’  3’-TGTGAGAAAGGGATGTGCTGCGAGAAGGCTAGp-5’ |
| 32. PE Adapter2 | 5’-pGATCGGAAGAGCGGTTCAGCAGGAATGCCGAG-3’  3’-TCTAGCCTTCTCGCCAAGTCGTCCTTACGGCTC-5’ |
| 33. PE PCR Primer1 | 5’-AATGATACGGCGACCACCGAGATCTACACTCTTTCCCTACACGACGCTCTTCCGATCT-3’ |
| 34. PE PCR Primer2 | 5’-CAAGCAGAAGACGGCATACGAGATCGGTCTCGGCATTCCTGCTGAACCGCTCTTCCGATCT-3’ |
| 35. Sequencing Primer | 5’-ACACTCTTTCCCTACACGACGCTCTTCCGATCT-3’ |
