# Supplementary material for: Mutagenic and Cytotoxic Properties of Oxidation Products of 5-Methylcytosine Revealed by Next-Generation Sequencing
Source: PLoS One. 2013 Sep 16;8(9):e72993. doi: 10.1371/journal.pone.0072993 (PMC3774748; doi:10.1371/journal.pone.0072993)
Supplement: Table S2 — The number of reads obtained by NGS for XG sequences. (DOC) [file pone.0072993.s004.doc]

### Table S2. The number of reads obtained by NGS for XG sequences.

| *E.coli* strains (triplicate experiments) | Cytosine derivative-containing genome | Total | A | C | G | T | A% | C% | G% | T% |
| --- | --- | --- | --- | --- | --- | --- | --- | --- | --- | --- |
| WT-1 | 5-hmC-XG | 3544 | 2 | 3534 | 0 | 8 | 0.056 | 99.72 | 0.000 | 0.226 |
|  | 5-foC-XG | 4112 | 0 | 4094 | 0 | 18 | 0.000 | 99.56 | 0.000 | 0.438 |
|  | 5-caC-XG | 3618 | 0 | 3579 | 0 | 39 | 0.000 | 98.92 | 0.000 | 1.078 |
|  | Control-CG | 4036 | 0 | 4034 | 2 | 0 | 0.000 | 99.95 | 0.050 | 0.000 |
| WT-2 | 5-hmC-XG | 3768 | 0 | 3750 | 0 | 18 | 0.000 | 99.52 | 0.000 | 0.478 |
|  | 5-foC-XG | 3424 | 0 | 3420 | 0 | 4 | 0.000 | 99.88 | 0.000 | 0.117 |
|  | 5-caC-XG | 4740 | 0 | 4710 | 0 | 30 | 0.000 | 99.37 | 0.000 | 0.633 |
|  | Control-CG | 4170 | 0 | 4170 | 0 | 0 | 0.000 | 100.00 | 0.000 | 0.000 |
| WT-3 | 5-hmC-XG | 4775 | 0 | 4770 | 0 | 5 | 0.000 | 99.90 | 0.000 | 0.105 |
|  | 5-foC-XG | 4270 | 0 | 4260 | 0 | 10 | 0.000 | 99.77 | 0.000 | 0.234 |
|  | 5-caC-XG | 4278 | 0 | 4252 | 0 | 26 | 0.000 | 99.39 | 0.000 | 0.608 |
|  | Control-CG | 3960 | 0 | 3960 | 0 | 0 | 0.000 | 100.00 | 0.000 | 0.000 |
| *Δ*pol II-1 | 5-hmC-XG | 4511 | 0 | 4505 | 2 | 4 | 0.000 | 99.87 | 0.044 | 0.089 |
|  | 5-foC-XG | 4443 | 0 | 4437 | 2 | 4 | 0.000 | 99.86 | 0.045 | 0.090 |
|  | 5-caC-XG | 3005 | 0 | 2981 | 1 | 23 | 0.000 | 99.20 | 0.033 | 0.765 |
|  | Control-CG | 3826 | 0 | 3824 | 0 | 2 | 0.000 | 99.95 | 0.000 | 0.052 |
| *Δ*pol II-2 | 5-hmC-XG | 3252 | 0 | 3237 | 0 | 15 | 0.000 | 99.54 | 0.000 | 0.461 |
|  | 5-foC-XG | 3903 | 3 | 3888 | 0 | 12 | 0.077 | 99.62 | 0.000 | 0.307 |
|  | 5-caC-XG | 3894 | 0 | 3834 | 0 | 60 | 0.000 | 98.46 | 0.000 | 1.541 |
|  | Control-CG | 3762 | 0 | 3762 | 0 | 0 | 0.000 | 100.00 | 0.000 | 0.000 |
| *Δ*pol II-3 | 5-hmC-XG | 3429 | 0 | 3420 | 0 | 9 | 0.000 | 99.74 | 0.000 | 0.262 |
|  | 5-foC-XG | 2646 | 0 | 2637 | 0 | 9 | 0.000 | 99.66 | 0.000 | 0.340 |
|  | 5-caC-XG | 3474 | 0 | 3456 | 0 | 18 | 0.000 | 99.48 | 0.000 | 0.518 |
|  | Control-CG | 4185 | 0 | 4185 | 0 | 0 | 0.000 | 100.00 | 0.000 | 0.000 |
| *Δ*pol IV-1 | 5-hmC-XG | 4998 | 0 | 4980 | 0 | 18 | 0.000 | 99.64 | 0.000 | 0.360 |
|  | 5-foC-XG | 4977 | 0 | 4965 | 0 | 12 | 0.000 | 99.76 | 0.000 | 0.241 |
|  | 5-caC-XG | 5784 | 0 | 5688 | 6 | 90 | 0.000 | 98.34 | 0.104 | 1.556 |
|  | Control-CG | 4860 | 0 | 4860 | 0 | 0 | 0.000 | 100.00 | 0.000 | 0.000 |
| *Δ*pol IV-2 | 5-hmC-XG | 5545 | 2 | 5538 | 1 | 4 | 0.036 | 99.87 | 0.018 | 0.072 |
|  | 5-foC-XG | 5169 | 0 | 5159 | 0 | 10 | 0.000 | 99.81 | 0.000 | 0.193 |
|  | 5-caC-XG | 5201 | 0 | 5135 | 3 | 63 | 0.000 | 98.73 | 0.058 | 1.211 |
|  | Control-CG | 5622 | 0 | 5614 | 4 | 4 | 0.000 | 99.86 | 0.071 | 0.071 |
| *Δ*pol IV-3 | 5-hmC-XG | 3630 | 0 | 3609 | 0 | 21 | 0.000 | 99.42 | 0.000 | 0.579 |
|  | 5-foC-XG | 3933 | 3 | 3927 | 0 | 3 | 0.076 | 99.85 | 0.000 | 0.076 |
|  | 5-caC-XG | 3735 | 0 | 3720 | 0 | 15 | 0.000 | 99.60 | 0.000 | 0.402 |
|  | Control-CG | 4059 | 3 | 4050 | 3 | 3 | 0.074 | 99.78 | 0.074 | 0.074 |
| *Δ*pol V-1 | 5-hmC-XG | 5132 | 0 | 5124 | 3 | 5 | 0.000 | 99.84 | 0.058 | 0.097 |
|  | 5-foC-XG | 5223 | 0 | 5208 | 0 | 15 | 0.000 | 99.71 | 0.000 | 0.287 |
|  | 5-caC-XG | 3084 | 0 | 3039 | 0 | 45 | 0.000 | 98.54 | 0.000 | 1.459 |
|  | Control-CG | 4788 | 0 | 4788 | 0 | 0 | 0.000 | 100.00 | 0.000 | 0.000 |
| *Δ*pol V-2 | 5-hmC-XG | 5988 | 2 | 5954 | 1 | 31 | 0.033 | 99.43 | 0.017 | 0.518 |
|  | 5-foC-XG | 4927 | 0 | 4917 | 0 | 10 | 0.000 | 99.80 | 0.000 | 0.203 |
|  | 5-caC-XG | 5257 | 1 | 5214 | 1 | 41 | 0.019 | 99.18 | 0.019 | 0.780 |
|  | Control-CG | 4889 | 1 | 4883 | 4 | 1 | 0.020 | 99.88 | 0.082 | 0.020 |
| *Δ*pol V-3 | 5-hmC-XG | 3580 | 0 | 3575 | 0 | 5 | 0.000 | 99.86 | 0.000 | 0.140 |
|  | 5-foC-XG | 3545 | 0 | 3540 | 0 | 5 | 0.000 | 99.86 | 0.000 | 0.141 |
|  | 5-caC-XG | 4870 | 0 | 4860 | 0 | 10 | 0.000 | 99.79 | 0.000 | 0.205 |
|  | Control-CG | 4052 | 2 | 4050 | 0 | 0 | 0.049 | 99.95 | 0.000 | 0.000 |
| *Δ*pol IV,V-1 | 5-hmC-XG | 5605 | 0 | 5600 | 0 | 5 | 0.000 | 99.91 | 0.000 | 0.089 |
|  | 5-foC-XG | 3404 | 0 | 3400 | 0 | 4 | 0.000 | 99.88 | 0.000 | 0.118 |
|  | 5-caC-XG | 4508 | 0 | 4464 | 0 | 44 | 0.000 | 99.02 | 0.000 | 0.976 |
|  | Control-CG | 4400 | 0 | 4400 | 0 | 0 | 0.000 | 100.00 | 0.000 | 0.000 |
| *Δ*pol IV,V-2 | 5-hmC-XG | 4890 | 0 | 4870 | 0 | 20 | 0.000 | 99.59 | 0.000 | 0.409 |
|  | 5-foC-XG | 5895 | 0 | 5870 | 0 | 25 | 0.000 | 99.58 | 0.000 | 0.424 |
|  | 5-caC-XG | 4005 | 0 | 3975 | 0 | 30 | 0.000 | 99.25 | 0.000 | 0.749 |
|  | Control-CG | 4500 | 0 | 4500 | 0 | 0 | 0.000 | 100.00 | 0.000 | 0.000 |
| *Δ*pol IV,V-3 | 5-hmC-XG | 3825 | 0 | 3819 | 0 | 6 | 0.000 | 99.84 | 0.000 | 0.157 |
|  | 5-foC-XG | 3595 | 0 | 3587 | 0 | 8 | 0.000 | 99.78 | 0.000 | 0.223 |
|  | 5-caC-XG | 4470 | 0 | 4430 | 0 | 40 | 0.000 | 99.11 | 0.000 | 0.895 |
|  | Control-CG | 3845 | 0 | 3844 | 0 | 1 | 0.000 | 99.97 | 0.000 | 0.026 |
