# Supplementary material for: Mutagenic and Cytotoxic Properties of Oxidation Products of 5-Methylcytosine Revealed by Next-Generation Sequencing
Source: PLoS One. 2013 Sep 16;8(9):e72993. doi: 10.1371/journal.pone.0072993 (PMC3774748; doi:10.1371/journal.pone.0072993)
Supplement: Table S3 — The number of reads obtained by NGS for XA sequences. (DOC) [file pone.0072993.s005.doc]

**Table S3.** The number of reads obtained by NGS for XA sequences.

| *E.coli* strains (triplicate experiments) | Cytosine derivative-containing genome | Total | A | C | G | T | A% | C% | G% | T% |
| --- | --- | --- | --- | --- | --- | --- | --- | --- | --- | --- |
| WT-1 | 5-hmC-XA | 3795 | 1 | 3784 | 0 | 10 | 0.026 | 99.71 | 0.000 | 0.264 |
|  | 5-foC-XA | 4388 | 2 | 4379 | 1 | 6 | 0.046 | 99.79 | 0.023 | 0.137 |
|  | 5-caC-XA | 3911 | 0 | 3875 | 0 | 36 | 0.000 | 99.08 | 0.000 | 0.920 |
|  | Control-CA | 4391 | 0 | 4390 | 0 | 1 | 0.000 | 99.98 | 0.000 | 0.023 |
| WT-2 | 5-hmC-XA | 3997 | 0 | 3981 | 0 | 16 | 0.000 | 99.60 | 0.000 | 0.400 |
|  | 5-foC-XA | 3740 | 0 | 3736 | 0 | 4 | 0.000 | 99.89 | 0.000 | 0.107 |
|  | 5-caC-XA | 3959 | 0 | 3921 | 0 | 38 | 0.000 | 99.04 | 0.000 | 0.960 |
|  | Control-CA | 3404 | 2 | 3401 | 0 | 1 | 0.059 | 99.91 | 0.000 | 0.029 |
| WT-3 | 5-hmC-XA | 3544 | 2 | 3534 | 0 | 8 | 0.056 | 99.72 | 0.000 | 0.226 |
|  | 5-foC-XA | 4112 | 0 | 4094 | 0 | 18 | 0.000 | 99.56 | 0.000 | 0.438 |
|  | 5-caC-XA | 3618 | 0 | 3579 | 0 | 39 | 0.000 | 98.92 | 0.000 | 1.078 |
|  | Control-CA | 4036 | 0 | 4034 | 2 | 0 | 0.000 | 99.95 | 0.050 | 0.000 |
| *Δ*pol II-1 | 5-hmC-XA | 5315 | 1 | 5285 | 0 | 29 | 0.019 | 99.44 | 0.000 | 0.546 |
|  | 5-foC-XA | 6575 | 0 | 6564 | 0 | 11 | 0.000 | 99.83 | 0.000 | 0.167 |
|  | 5-caC-XA | 6903 | 1 | 6844 | 0 | 58 | 0.014 | 99.15 | 0.000 | 0.840 |
|  | Control-CA | 5561 | 1 | 5559 | 0 | 1 | 0.018 | 99.96 | 0.000 | 0.018 |
| *Δ*pol II-2 | 5-hmC-XA | 4122 | 0 | 4120 | 0 | 2 | 0.000 | 99.95 | 0.000 | 0.049 |
|  | 5-foC-XA | 3776 | 0 | 3752 | 2 | 22 | 0.000 | 99.36 | 0.053 | 0.583 |
|  | 5-caC-XA | 3884 | 0 | 3844 | 0 | 40 | 0.000 | 98.97 | 0.000 | 1.030 |
|  | Control-CA | 4722 | 3 | 4716 | 0 | 3 | 0.064 | 99.87 | 0.000 | 0.064 |
| *Δ*pol II-3 | 5-hmC-XA | 4542 | 0 | 4521 | 0 | 21 | 0.000 | 99.54 | 0.000 | 0.462 |
|  | 5-foC-XA | 4725 | 0 | 4716 | 3 | 6 | 0.000 | 99.81 | 0.063 | 0.127 |
|  | 5-caC-XA | 3435 | 0 | 3384 | 0 | 51 | 0.000 | 98.52 | 0.000 | 1.485 |
|  | Control-CA | 4593 | 0 | 4590 | 0 | 3 | 0.000 | 99.93 | 0.000 | 0.065 |
| *Δ*pol IV-1 | 5-hmC-XA | 4389 | 0 | 4370 | 0 | 19 | 0.000 | 99.57 | 0.000 | 0.433 |
|  | 5-foC-XA | 4425 | 0 | 4406 | 0 | 19 | 0.000 | 99.57 | 0.000 | 0.429 |
|  | 5-caC-XA | 4935 | 0 | 4907 | 0 | 28 | 0.000 | 99.43 | 0.000 | 0.567 |
|  | Control-CA | 4995 | 2 | 4991 | 1 | 1 | 0.040 | 99.92 | 0.020 | 0.020 |
| *Δ*pol IV-2 | 5-hmC-XA | 3881 | 0 | 3851 | 2 | 28 | 0.000 | 99.23 | 0.052 | 0.721 |
|  | 5-foC-XA | 3280 | 0 | 3278 | 1 | 1 | 0.000 | 99.94 | 0.030 | 0.030 |
|  | 5-caC-XA | 4371 | 0 | 4342 | 0 | 29 | 0.000 | 99.34 | 0.000 | 0.663 |
|  | Control-CA | 3739 | 1 | 3736 | 0 | 2 | 0.027 | 99.92 | 0.000 | 0.053 |
| *Δ*pol IV-3 | 5-hmC-XA | 4147 | 0 | 4138 | 1 | 8 | 0.000 | 99.78 | 0.024 | 0.193 |
|  | 5-foC-XA | 4529 | 0 | 4507 | 1 | 21 | 0.000 | 99.51 | 0.022 | 0.464 |
|  | 5-caC-XA | 3338 | 0 | 3295 | 1 | 42 | 0.000 | 98.71 | 0.030 | 1.258 |
|  | Control-CA | 4087 | 1 | 4084 | 1 | 1 | 0.024 | 99.93 | 0.024 | 0.024 |
| *Δ*pol V-1 | 5-hmC-XA | 4430 | 0 | 4425 | 0 | 5 | 0.000 | 99.89 | 0.000 | 0.113 |
|  | 5-foC-XA | 4570 | 0 | 4560 | 0 | 10 | 0.000 | 99.78 | 0.000 | 0.219 |
|  | 5-caC-XA | 3945 | 0 | 3920 | 0 | 25 | 0.000 | 99.37 | 0.000 | 0.634 |
|  | Control-CA | 3450 | 0 | 3445 | 5 | 0 | 0.000 | 99.86 | 0.145 | 0.000 |
| *Δ*pol V-2 | 5-hmC-XA | 5029 | 0 | 5016 | 1 | 12 | 0.000 | 99.74 | 0.020 | 0.239 |
|  | 5-foC-XA | 6374 | 1 | 6339 | 0 | 34 | 0.016 | 99.45 | 0.000 | 0.533 |
|  | 5-caC-XA | 5531 | 0 | 5504 | 0 | 27 | 0.000 | 99.51 | 0.000 | 0.488 |
|  | Control-CA | 6949 | 0 | 6947 | 0 | 2 | 0.000 | 99.97 | 0.000 | 0.029 |
| *Δ*pol V-3 | 5-hmC-XA | 3584 | 0 | 3560 | 0 | 24 | 0.000 | 99.33 | 0.000 | 0.670 |
|  | 5-foC-XA | 3593 | 0 | 3588 | 0 | 5 | 0.000 | 99.86 | 0.000 | 0.139 |
|  | 5-caC-XA | 3928 | 0 | 3856 | 0 | 72 | 0.000 | 98.17 | 0.000 | 1.833 |
|  | Control-CA | 4760 | 0 | 4760 | 0 | 0 | 0.000 | 100.00 | 0.000 | 0.000 |
| *Δ*pol IV,V-1 | 5-hmC-XA | 4730 | 0 | 4725 | 0 | 5 | 0.000 | 99.89 | 0.000 | 0.106 |
|  | 5-foC-XA | 5505 | 0 | 5490 | 0 | 15 | 0.000 | 99.73 | 0.000 | 0.272 |
|  | 5-caC-XA | 5880 | 0 | 5820 | 0 | 60 | 0.000 | 98.98 | 0.000 | 1.020 |
|  | Control-CA | 4605 | 0 | 4605 | 0 | 0 | 0.000 | 100.00 | 0.000 | 0.000 |
| *Δ*pol IV,V-2 | 5-hmC-XA | 3495 | 0 | 3468 | 3 | 24 | 0.000 | 99.23 | 0.086 | 0.687 |
|  | 5-foC-XA | 4785 | 0 | 4770 | 0 | 15 | 0.000 | 99.69 | 0.000 | 0.313 |
|  | 5-caC-XA | 4704 | 0 | 4689 | 0 | 15 | 0.000 | 99.68 | 0.000 | 0.319 |
|  | Control-CA | 4911 | 0 | 4911 | 0 | 0 | 0.000 | 100.00 | 0.000 | 0.000 |
| *Δ*pol IV,V-3 | 5-hmC-XA | 4004 | 0 | 3988 | 0 | 16 | 0.000 | 99.60 | 0.000 | 0.400 |
|  | 5-foC-XA | 3724 | 0 | 3706 | 0 | 18 | 0.000 | 99.52 | 0.000 | 0.483 |
|  | 5-caC-XA | 3693 | 2 | 3669 | 0 | 22 | 0.054 | 99.35 | 0.000 | 0.596 |
|  | Control-CA | 4188 | 0 | 4186 | 0 | 2 | 0.000 | 99.95 | 0.000 | 0.048 |
